# A comparative study of interfacial thermal conductance between metal and semiconductor

**DOI:** 10.1038/s41598-022-24379-z

**Published:** 2022-11-19

**Authors:** Kongping Wu, Leng Zhang, Danbei Wang, Fangzhen Li, Pengzhan Zhang, Liwen Sang, Meiyong Liao, Kun Tang, Jiandong Ye, Shulin Gu

**Affiliations:** 1 School of Electronics and Information Engineering, Jinling Institute of Technology, Nanjing, 211169 Jiangsu China; 2 Research Center for Functional Materials, National Institute for Materials Science (NIMS), Tsukuba, Ibaraki 305-0044 Japan; 3 School of Electronic Science and Engineering, Nanjing University, Nanjing, 210093 Jiangsu China

**Keywords:** Surfaces, interfaces and thin films, Electronic properties and materials

## Abstract

To understand and control thermal conductance of interface between metal and semiconductor has now become a crucial task for the thermal design and management of nano-electronic and micro-electronic devices. The interfacial alignments and electronic characteristics of the interfaces between metal and semiconductor are studied using a first-principles calculation based on hybrid density functional theory. The thermal conductance of interfaces between metal and semiconductor were calculated and analyzed using diffuse mismatch model, acoustic mismatch model and nonequilibrium molecular dynamics methods. Especially, according to nonequilibrium molecular dynamics, the values of thermal conductance were obtained to be 32.55 MW m^−2^ K^−1^ and 341.87 MW m^−2^ K^−1^ at C–Cu and Si–Cu interfaces, respectively. These results of theoretical simulation calculations are basically consistent with the current experimental data, which indicates that phonon–phonon interaction play a more important role than electron–phonon interaction during heat transport. It may be effective way to improve the interfacial thermal conductance through enhancing the interface coupling strength at the metal–semiconductor interface because the strong interfacial scattering plays a role in suppressing in the weaker interface coupling heterostructure, leading to the lower thermal conductance of interfaces. This could provide a beneficial reference for the design of the Schottky diode and thermal management at the interfaces between metal and semiconductor.

## Introduction

With the rapid development of microelectronics technology, especially electronic devices are becoming smaller and smaller, high-power density devices have been widely developed and applied. When the power of electronic devices increases, their heat consumption also increases. If the heat consumption could not be dissipated in time, it would result in an increase in the failure rate and a decrease in reliability of semiconductor devices. It is crucial for this kind of electronic devices that constituent semiconductors and the interface between the constituent semiconductors have a good heat dissipation performance. Because both constituent semiconductors and the interface can scatter phonons and induce a lower thermal conductivity. Diamond (C) with a high breakdown field strength (> 10 MV cm^−1^)^1^ and a high thermal conductivity (> 2000 W m^−1^ K^−1^)^2^ has recently attracted tremendous attention and research interest due to its promising potential applications in high-power switches^3,4^, high-frequency field-effect transistors^5,6^ and Schottky diodes^7,8^.

Although it is a broad consensus that diamond has very high thermal conductivity, the interfacial thermal conductance (*G*) at the interface between diamond and other materials is not easy to determine. Recently, the result of *G* studies at GaN/diamond interface show a higher value (90–128.2 MW m^−2^ K^−1^)^9,10^, while some research results of G at metal/diamond interfaces are very low (4.18 MW m^−2^ K^−1^ for Cu/diamond, 0.31 MW m^−2^ K^−1^ for Ni/diamond)^11,12^. And especially, for the interfaces between metals and diamond, the results of *G* are also widely various^13–15^. The various factors are directly or indirectly affecting the value of *G*, such as interface roughness^16^, bond strain^17^, surface termination of the semiconductor^18^ and interfacial defects^19^ and so on.

At present, the acoustic mismatch model (AMM) and diffuse mismatch model (DMM) are considered as two standard models to describe phonon scattering^20–22^. Especially, in the DMM, when the phonons arrive at the interface, scattering and transmission occur, and the transmission probability is determined by the phonon density of states (Ph-DOS) on both sides of the interface, which is independent of the Ph-DOS before transmission^23–25^. Besides, non-equilibrium molecular dynamics (NEMD) method was employed to study the *G* by establishing a temperature gradient across the interfaces between metal and diamond. In this method, two groups of atoms at both ends are fixed as heat source and heat sink, respectively. Where the temperature jump caused by the interface is included, as well as the anharmonic interactions between atoms are involved^26,27^.

In this work, the *G* was calculated for the four types of interfaces: Ni–Si, Ni–C, Cu–Si and Cu–C. In these interfaces, Ni and Cu have similar crystal structure and are important transition metals used as electrodes^28^. Diamond and silicon have similar crystal structure and are composed of one element C and one element Si, respectively. Here, the density functional theory calculation method is used to calculate the electronic and phonon properties of these interfaces. First, the interfacial interaction and stability of the these interfaces with different interfacial configurations are calculated and discussed, and then the electronic properties of the stable interface are calculated, including charge transfer at these interfaces, band alignment and the Schottky barrier height of the metal–semiconductor contact. Subsequently, phonons are served as heat carriers in the process of thermal transport at these interfaces. According to the density functional perturbation theory, the second-order force constant was obtained under the harmonic approximation. According to the mismatch models (the DMM and AMM) and NEMD methods, the *G* of these interfaces were calculated and analyzed. Finally, the theoretical results are compared with the experimental data to explore the heat transport mechanism behind the interfacial thermal conductance. These findings provide a theoretical basis for a thorough understanding of the electronic properties and thermal transport characteristics of the metal/semiconductor interfaces, and provide a theoretical reference for the preparation of power device based on the metal/semiconductor Schottky diodes.

## Models and computational details

The Vienna Ab-initio Simulation software Package (VASP) based on density functional theory is used to calculate the physical properties of these interfaces, and the projection plane wave pseudopotential is used to describe the relationship between the ion core and the valence electron. For the interaction potential, the spatial non-local exchange correlation function (Heyd, Scuseria and Ernzerhof, HSE hybrid functional) method is used to deal with the interaction between electrons^29,30^. The matrix element of the Hamiltonian is determined by the plane wave expansion with the charge density and the Kohn–Sham potential function with cut-off energy of 500 eV, and convergence standard of 10^–5^ eV in electronic self-consistent iterative loop is used. When calculating the Ni–C and Cu–C interfaces, the diamond surface model is cleaved along the [100] direction of the diamond crystal with in-plane lattice vectors [010] and [001] directions. And for Ni–Si and Cu–Si interfaces, the Si surface model is cleaved along the [001] plane of the Si crystal with in-plane lattice vectors [110]a_0_/2 and [1-1-10]a_0_/2 orientations. These interface systems contain six C or Si atomic layers, five Ni or Cu atomic layers, and a vacuum layer with a thickness of 15 Å. After testing, the error in energy calculation is controlled within 0.1 eV.

For NEMD methods, both Cu–C and Cu–Si interfaces were simulated by molecular dynamics simulation package, LAMMPS. And modified embedded-atom method (MEAM) interatomic potentials are employed for the Cu–C and Cu–Si interfaces^31,32^. In this work, the temperature of the heat source and the heat sink were set to 320 K and 280 K, respectively. To ensure that the temperature gradient has a reasonable value. After the system reaches steady state, the temperature gradient is measured, and then the thermal conductance is calculated according to Fourier law^33^.

In the structure optimization of these interfaces, the conjugate gradient minimization scheme is used to optimize the structure of each model system. A 7 × 7 × 1 Brillouin zone grid is used in the optimization of each interface model, and a matched irreducible K point scheme is automatically generated through Monkhorst-Pack^34^. Finally, the force of each atom in the system is less than 10^–3^ eV/Å. In addition, the Brillouin zone grid of 13 × 13 × 1 was used to calculate the density of states and potential function. Besides, the force constants under the quasi-harmonic approximation were calculated for a 2 × 2 × 2 Si or diamond supercell with 16 Si or C atoms and a 3 × 3 × 3 Ni or Cu supercell with 27 Ni or Cu atoms. Density functional perturbation theory (DFPT) calculation was performed^35^, the force constant was obtained by the finite difference method for each system.

**Figure 1:**
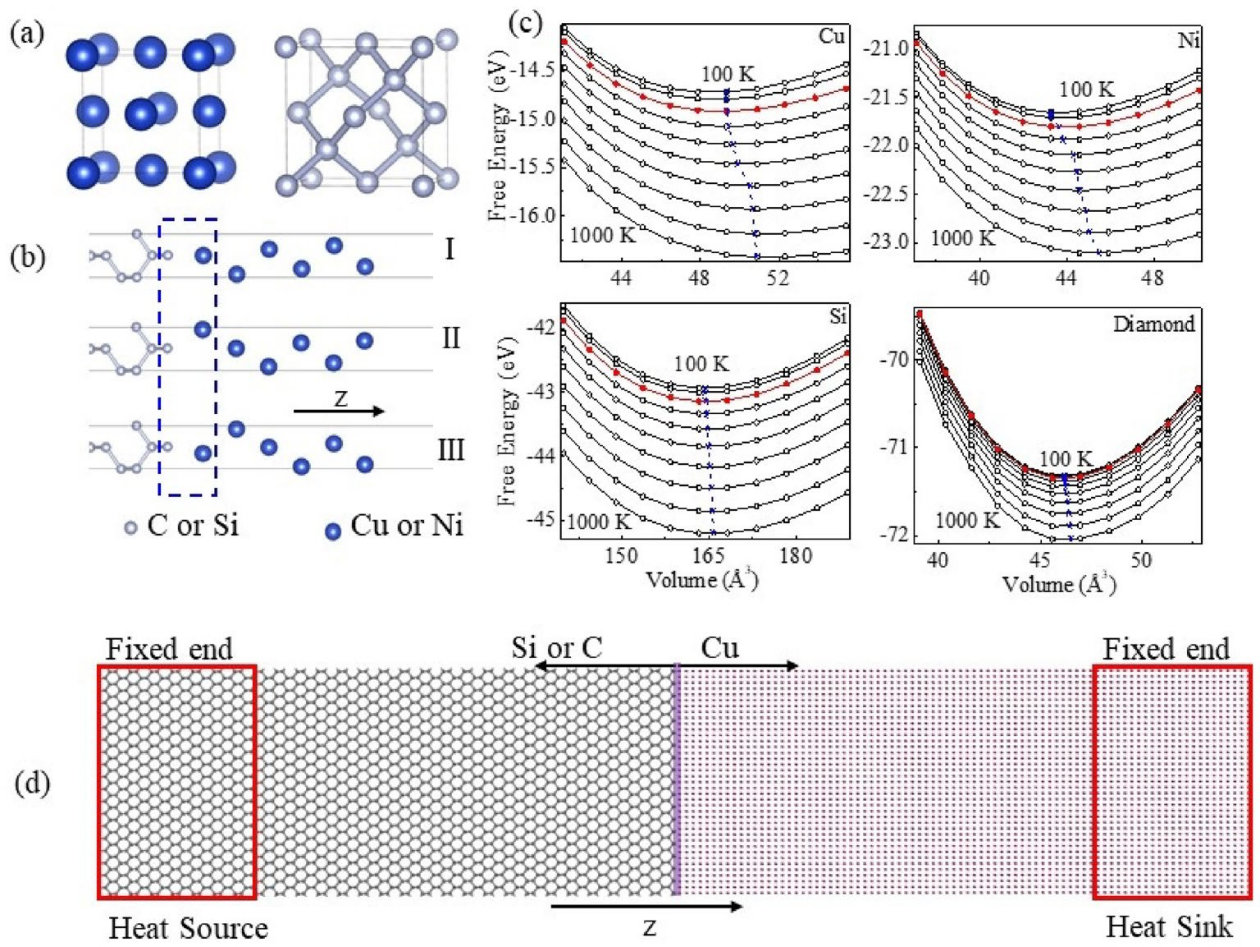
(**a**) Lattice structures of metal Ni or Cu and Si or diamond (left and top). (**b**) Type I, Type II and Type III for Ni–Si interface (left and bottom). (**c**) Relationship for Cu, Ni, Si and diamond between free energy and unit cell volume from 100 to 1000 K with a step of 100 K, respectively. (Right) And (**d**) Side view of the Cu–C and Cu–Si interfaces. The red rectangles with the width of 5 nm indicate heat source and sink regions, respectively, during NEMD simulation.

For the Ni–Si interface, it was constructed by three different alignment methods as shown in Fig. 1. Here, the interfacial stability of the Ni–Si interface can be quantitatively assessed by their interface formation energy. The interface formation energy is defined as^27,36^

1 $$ {\text{E}}_{{{\text{form}}}} = {\text{ E}}_{{{\text{Ni}} - {\text{Si}}}} {-}{\text{E}}_{{\text{Si-slab}}} {-}{\text{E}}_{{\text{Ni-slab}}} , $$

where E_Ni–Si_ is the total energy of the Ni–Si interface structure, E_Si-slab_ and E_Ni-slab_ are the total energy of the part of Si and Ni in these interfaces, respectively. Finally, the Ni–Si interface with the negative E_form_ and the larger absolute value is regarded as the most stable interface. The stable structures of the other three interfaces are determined by the similar method.

The *G* of every interface was determined according to the Landauer transport equation^37^, which can be written as:

2 $$ G = \frac{{1}}{{2}} \cdot \frac{{1}}{{2}}\sum\nolimits_{\bmod e - i1}^{\bmod e - in} {\int_{0}^{\omega } {V\alpha_{1 \to 2} } } \cdot (\hbar \omega ) \cdot \frac{dN}{{dT}}d\omega , $$

where V is the group velocity of the phonon, and the group velocity of the phonon can be written as: $V = 2\pi \frac{\partial f}{{\partial k}}$, and $\omega = 2\pi f$.

The parameter $\alpha_{1 \to 2}$ is the transmission of phonons from the Si or diamond to the metal Ni or Cu. Combining the phonon group velocity and density of states of each vibration mode in the two materials, the transmission of phonons can be written as the following form:

3 $$ \alpha_{1 \to 2} = \frac{{\sum\nolimits_{\bmod e - j1}^{\bmod e - jn} {DOS_{2,j} \cdot V_{2,j} } }}{{\sum\nolimits_{\bmod e - k1}^{\bmod e - kn} {DOS_{1,k} \cdot V_{1,k} } + \sum\nolimits_{\bmod e - j1}^{\bmod e - jn} {DOS_{2,j} \cdot V_{2,j} } }}, $$

where N is the number of phonons: $N = DOS \cdot f_{B - E} = \frac{DOS}{{\exp \left( {\frac{\hbar \omega }{{k_{B} T}}} \right) - 1}}$, and $f_{B - E}$ is the Bose–Einstein distribution, $k_{B}$ is the Boltzmann constant. After taking the derivative with respect to the temperature (T) on the number of phonons (N), it can be written as:

4 $$ \frac{dN}{{d{\text{T}}}} = DOS \cdot \frac{\hbar \omega }{{k_{B} T^{2} }} \cdot \frac{{\exp \left( {\frac{\hbar \omega }{{k_{B} T}}} \right)}}{{\left[ {\exp \left( {\frac{\hbar \omega }{{k_{B} T}}} \right) - 1} \right]^{2} }}. $$

Therefore, the final interface thermal conductance (*G*) can be written as:

5 $$ G = \frac{{1}}{{2}} \cdot \frac{{1}}{{2}}\sum\nolimits_{\bmod e - i1}^{\bmod e - in} {\int_{0}^{\omega } {DOS \cdot V\alpha_{1 \to 2} } } \cdot (\hbar \omega ) \cdot \frac{\hbar \omega }{{k_{B} T^{2} }} \cdot \frac{{\exp \left( {\frac{\hbar \omega }{{k_{B} T}}} \right)}}{{\left[ {\exp \left( {\frac{\hbar \omega }{{k_{B} T}}} \right) - 1} \right]^{2} }}d\omega . $$

## Results and discussion

### The characteristics of the equilibrium structures

The equilibrium lattice constants a and bulk moduli B_0_ were calculated using VASPKIT, which is realized by fitting the Birch–Murnaghan equation of state^38,39^. The obtained total energy of Cu, Ni, Si and diamond as a function of lattice constants were shown in Fig. S1 in Supplementary Material, and the calculated equilibrium lattice constants and bulk moduli B_0_ and the available experimental data of Cu, Ni, Si and diamond were summarized in Table 1, respectively. The calculated lattice parameter is 3.511 Å for Ni, which is lightly underestimated in comparison to the experimental data^40^. While the other three, 3.631 Å for Cu, 5.465 Å for Si and 3.572 Å for diamond, are lightly overestimated in comparison to the experimental data. The calculated bulk moduli B_0_ are 136.31, 197.71, 88.87 and 433.55 GPa for Cu, Ni, Si and diamond, respectively. These calculated results are also much closer to the experiment as shown in Table 1. In addition, the calculated band gaps of Si and diamond are about 1.17 and 5.38 eV, which are basically consistent with the experimental values (1.12 and 5.47 eV).

**Table 1:** The calculated lattice constants and bulk moduli B_0_, in comparison with experiments.

Material	Lattice constants a (Å)		Bulk moduli B_0_ (GPa)	
	This work	Experiments	This work	Experiments
Cu	3.631 (3.645)	3.624^41^	136.31	133^42^
Ni	3.511 (3.522)	3.523^40^	197.71	186–195^43^
Si	5.435 (5.454)	5.43^44,45^	88.87	98.8^45^
Diamond	3.572 (3.578)	3.567^46^	433.55	443^47^

And the calculated lattice constants at room temperature were also listed in parentheses.

For the purpose of comparisons between the calculated results and experimental data for the *G* at room temperature, the equilibrium lattice constants of Cu, Ni, Si and diamond at room temperature were also considered. According to the first principles calculation and the quasi-harmonic Debye–Gruneisen approximation theory^48^, the temperature-dependent elastic modulus of Cu, Ni, Si and diamond were taken into account, respectively. And the Birch–Murnaghan equation of state was used to determine the equilibrium dependence between energy, volume and temperature as shown in Fig. 1c. The equilibrium lattice constants of Cu, Ni, Si and diamond at room temperature are determined to be 3.645, 3.522, 5.454 and 3.578 Å, respectively.

When establishing the interface, the in-plane lattice constant has been fixed by the lattice constant of semiconductor, because the metal has good ductility. The stress caused by strain is basically imposed on the metal. The Si [001] plane has a square lattice with in-plane constants of 3.847 Å for [1$\overline{1 }$0] and [110] orientations. The lattice mismatch in Ni–Si, Ni–C, Cu–Si and Cu–C interfaces are − 8.68%, − 1.7%, − 5.25% and 2.03%, respectively. Taking the Cu–C interface as an example, the Cu–C interface structure adopts three different alignment modes of C atoms and Cu atoms above the diamond surface as shown in Fig. 1b.

According to the equation of the interface formation energy, we can obtain that the interface formation energy is about − 1.11 eV for the type I, the interface formation energy for the type II is about − 1.88 eV, and the interface formation energy for the type III is about − 1.81 eV. The type II is more stable with lower interface formation energy than the types I and III, which is entirely consistent with a recent theoretical calculation result^49^. And the calculated interface distance (the interplanar distance along the z direction between C and Cu) and the minimum interatomic distance between C and Cu are about 1.445 and 1.997 Å for the type II and listed in Table 2. Based on these interface parameters, C–Cu and Si–Cu heterostructures with the length of 305.76 Å and 303.16 Å along z direction were built as show in Fig. 1d, respectively. During the NEMD simulation, a non-equilibrium steady state established, the heat flux from heat source to heat sink is time independent, and a temperature jump (ΔT) is induced at the interface due to the interfacial thermal resistance.

**Table 2:** The key parameters of the Ni–Si, Ni–C, Cu–Si and Cu–C interfaces after optimization.

Interfaces	Interface distance (Å)	Minimum atomic distance at interface (Å)	Interatomic distance at semiconductor surface (Å)	Interface formation energy (eV)	Sum of charge transfer (e/C)	Schottky barrier height ϕ_B_ (eV)
Cu–C	1.445	1.997	1.524	− 1.88	0.26	1.57 (BLM) 1.80 (IIM)
						1.60 Ref.^61^
Ni–C	1.457	1.918	1.513	− 0.45	0.28	1.45 (BLM) 1.81 (IIM)
						1.60 Ref.^62^
Cu–Si	1.499	2.831	2.354	− 0.23	0.01	0.66 (BLM) 0.56 (IIM)
						0.51 Ref.^63^
Ni–Si	1.485	2.721	2.337	− 0.17	0.04	0.61 (BLM) 0.57 (IIM)
						0.61 Ref.^64^

The part of key parameters (interface distance, the minimum interatomic distance and lower interface formation energy) in the Ni–Si, Ni–C and Cu–Si interfaces were calculated similarly to those obtained in Cu–C interface and also shown in Table 2. Besides, interatomic distances (C–C and Si–Si) at the first layer of semiconductor (d_s–s_) were also calculated and shown in Table 2 as a reference for interface distance and interatomic distances. As shown in Table 2, it is found that the interface distance and the interface formation energy of Cu–C, Ni–C, Ni–Si and Cu–Si interfaces are in turn increase, and implies that in descending order of the interfacial interaction strength are: Cu–C, Ni–C, Ni–Si and Cu–Si interfaces^12,27^. The low formation energy normally means a stable interfacial configuration. And the lower interface formation energy also predicts a stronger interfacial bonding, which can increase phonon transfer across the interface and further improve the thermal conductance at the interface. In addition, the values of Pauling’s electronegativity of the elements Cu, Ni, Si and C are 1.90, 1.91, 1.90 and 2.55, respectively^50–52^. The electronegativity difference between the C and the metals (Cu and Ni) is large compared to the electronegativity difference between the Si and metals in these interfaces, resulting in more charge transfer from metal to C and larger interface interaction between the C and the metals.

### Electronic properties of these interfaces

In order to further explore the charge transfer at these interfaces, Bader charges of all atoms near the interface was calculated and analyzed^53,54^. According to our calculation, the charge transfer only occurred at the interface. And the average value of charge transfer for each atom at the interface was listed in Table 2. It can be found that the C at the interface obtains more charge (about 0.27 ± 0.01e per C atom) from the metal layer than Si at the interface (about 0.01–0.04e per Si atom). Specifically, on the side of the diamond at the Cu–C interface, the charge obtained is 0.26e per atom for the interfacial C atomic. For the Ni–C interface, the amount of charge transfer is basically the same. In contrast, there is almost no charge transfer at the Ni–Si and Cu–Si interfaces.

**Figure 2:**
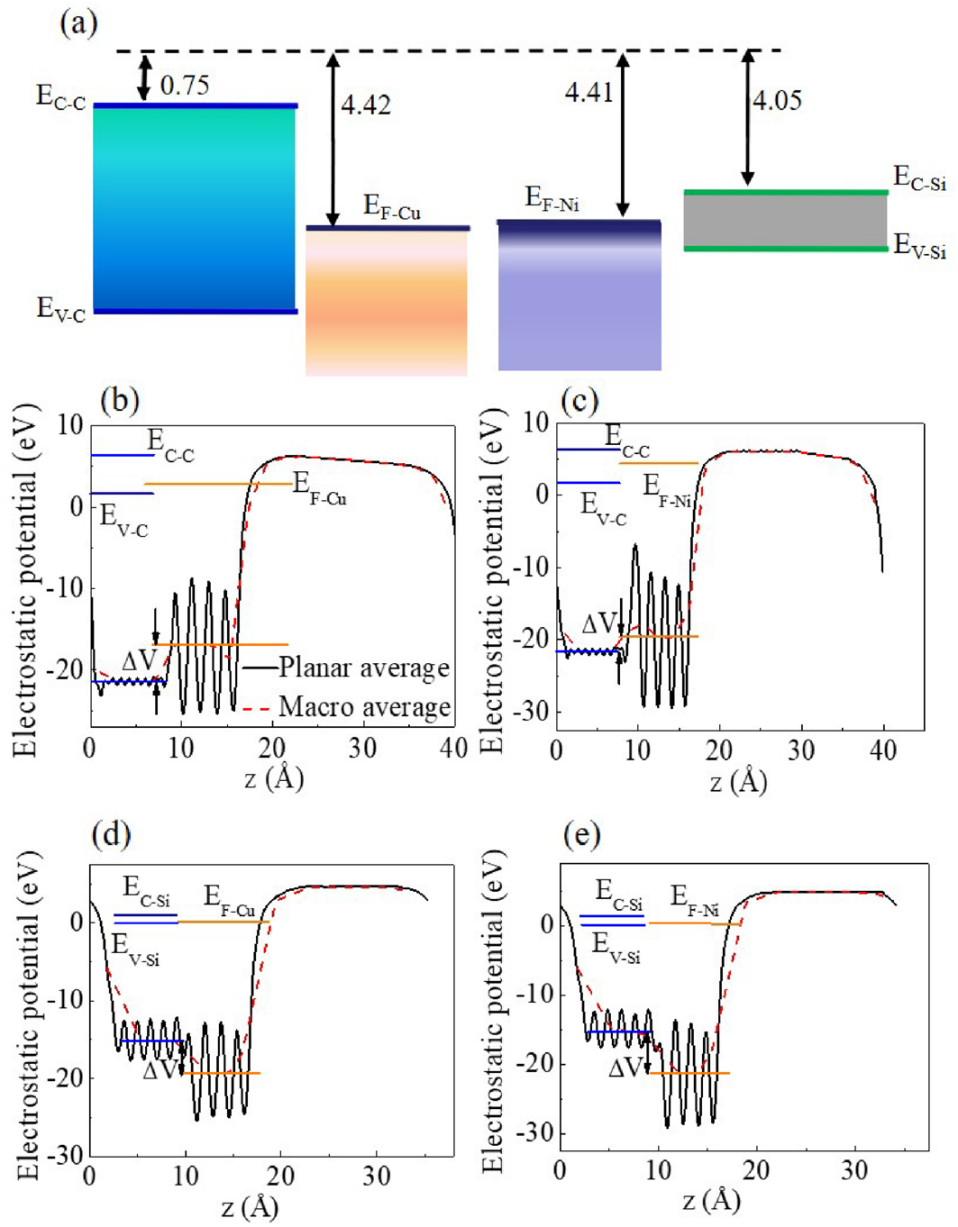
(**a**) Energy band alignment for the Cu, Si, Ni and diamond according to the experimental values of electron affinity and work function. Planar and macroscopic average of the electrostatic potential as a function of the position along the z direction for (**b**) the Cu–C, (**c**) the Ni–C, (**d**) the Cu–Si, (**e**) the Ni–Si interfaces. The E_C–C_ and E_C–Si_ are the conduction band bottom and E_V–C_ and E_V–Si_ are valence band top for diamond and Si, respectively. The E_F–Cu_ and E_F–Ni_ are Fermi level of Cu and Ni, respectively. The ΔV is the difference of macroscopic average of the electrostatic potential for these interfaces.

The bending of the energy band of the semiconductor at the interface induces a Schottky barrier when the metal contacts with the semiconductor material. The role of the local chemistry and electronic properties can also be understood by the calculations of Schottky barrier (*Φ*_SB_). In an ideal interface model (IIM), *Φ*_SB_ can be calculated by the Schottky–Mott rule using energy-level alignments^55^. According to the work function of the metal (4.41 eV for Ni and 4.42 eV for Cu)^56^ and the electron affinity of the semiconductor (4.05 eV for Si and 0.75 eV for diamond)^57,58^, energy band alignment is shown in Fig. 2a, which predicts *Φ*_SB_ values of 1.80, 1.81, 0.56 and 0.57 eV for Cu–C (p type), Ni–C (p type), Ni–Si (n type) and Cu–Si (n type) interfaces, respectively.

Besides, the “bulk plus lineup” method (BLM)^59,60^ was used to calculate the *Φ*_SB_ as shown in Fig. 2b–e for the Cu–C, Ni–C, Cu–Si and Ni–Si interfaces, respectively. The planar and macroscopic average of the electrostatic potential were calculated for each interface, and the conduction band bottom (E_C_), valence band top (E_V_) for semiconductors and Fermi level for metals were determined in Fig. 2b–e. The calculated values of the *Φ*_SB_, 1.57 eV for Cu–C (p type), 1.45 eV for Ni–C (p type), 0.66 eV for Cu–Si (n type) and 0.61 eV for Ni–Si (n type), are listed in Table 2 and basically consistent with the experimental data of 1.60, 1.60, 0.62 and 0.56 eV for Cu–C (p type), Ni–C (p type), Cu–Si (n type) and Ni–Si (n type) interfaces^61–64^, respectively. And especially, compared with the interfaces based on diamond, the values of the *Φ*_SB_ for the interfaces based on Si is closer to that obtained by the ideal interface model, indicating a relatively weak coupling strength^55^.

### Thermal transport characteristics at these interfaces

**Figure 3:**
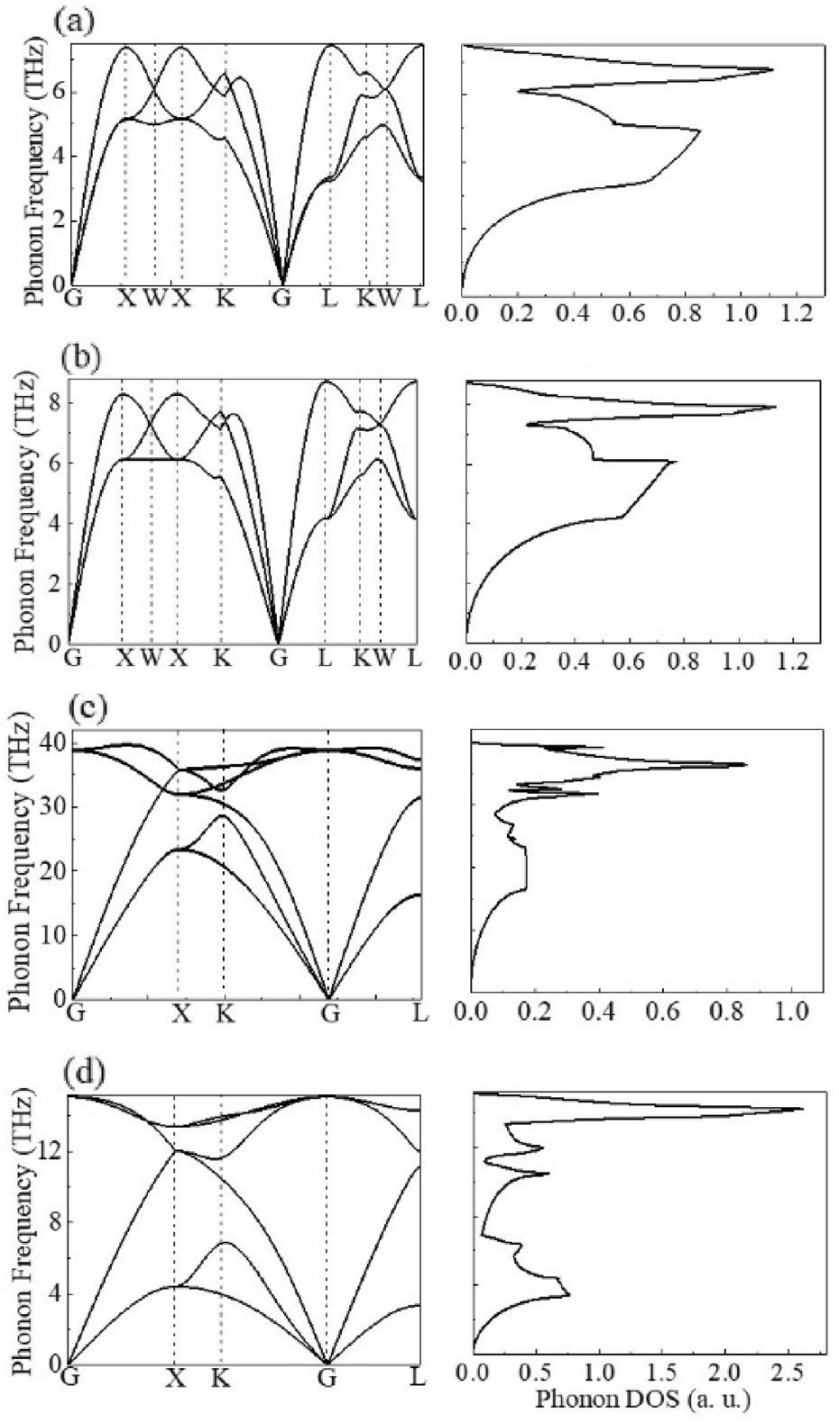
Phonon dispersion curves (left) and phonon DOS (right) for (**a**) Cu, (**b**) Ni, (**c**) diamond and (**d**) Si, respectively.

For heat transport at interface between metal and semiconductor, there are two possible pathways for heat transfer. The first is a coupling between phonons in metal and phonons in semiconductor (phonon–phonon). The second is a coupling between electrons in metal and phonons in semiconductor (electron–phonon). In this work, phonon was considered as the main carriers of heat transport, and the DFPT was used to calculate the lattice dynamics, where the second-order interatomic force constants were obtained for each unit cell under the harmonic approximation^65^. Figure 3a–d are the phonon dispersion curves and the corresponding phonon density of states (DOS) for Cu, Ni, diamond and Si, respectively. The calculated phonon dispersion and phonon DOS are in good agreement with the experimental data from the inelastic neutron scattering measurement^66–69^. Phonon DOS is essentially the number of phonons allowed to occupy in different states for a specific energy level. And the mode density of lattice vibration is a function of the frequency. From the frequency distribution of phonon DOS, the cut-off frequencies of Cu and Ni appears at about 7.2 and 8.8 THz, respectively. While the cut-off frequencies of diamond and Si extend to about 40 and 15.2 THz, respectively. The calculation results are in good agreement with the results reported in the experiment and other theoretical calculations^66–72^.

The frequency distributions of Cu and Ni are mostly in the range of 2.0–7.0 and 3.0–8.5 THz, while for diamond and Si the frequency ranges are mainly from 10 to 40 THz and from 4 to 14 THz, respectively. Compared to Si, the phonon frequency distribution of diamond extends to a wider range and more phonons carry energy. In addition, in the overlapping frequency range (below 10 THz) between metals and semiconductors, the phonon DOS of diamond is much lower than that of Si. Therefore, for ideal interfaces these could lead to a decrease of the elastic transport for the phonons through the interfaces based diamond^73^.

**Figure 4:**
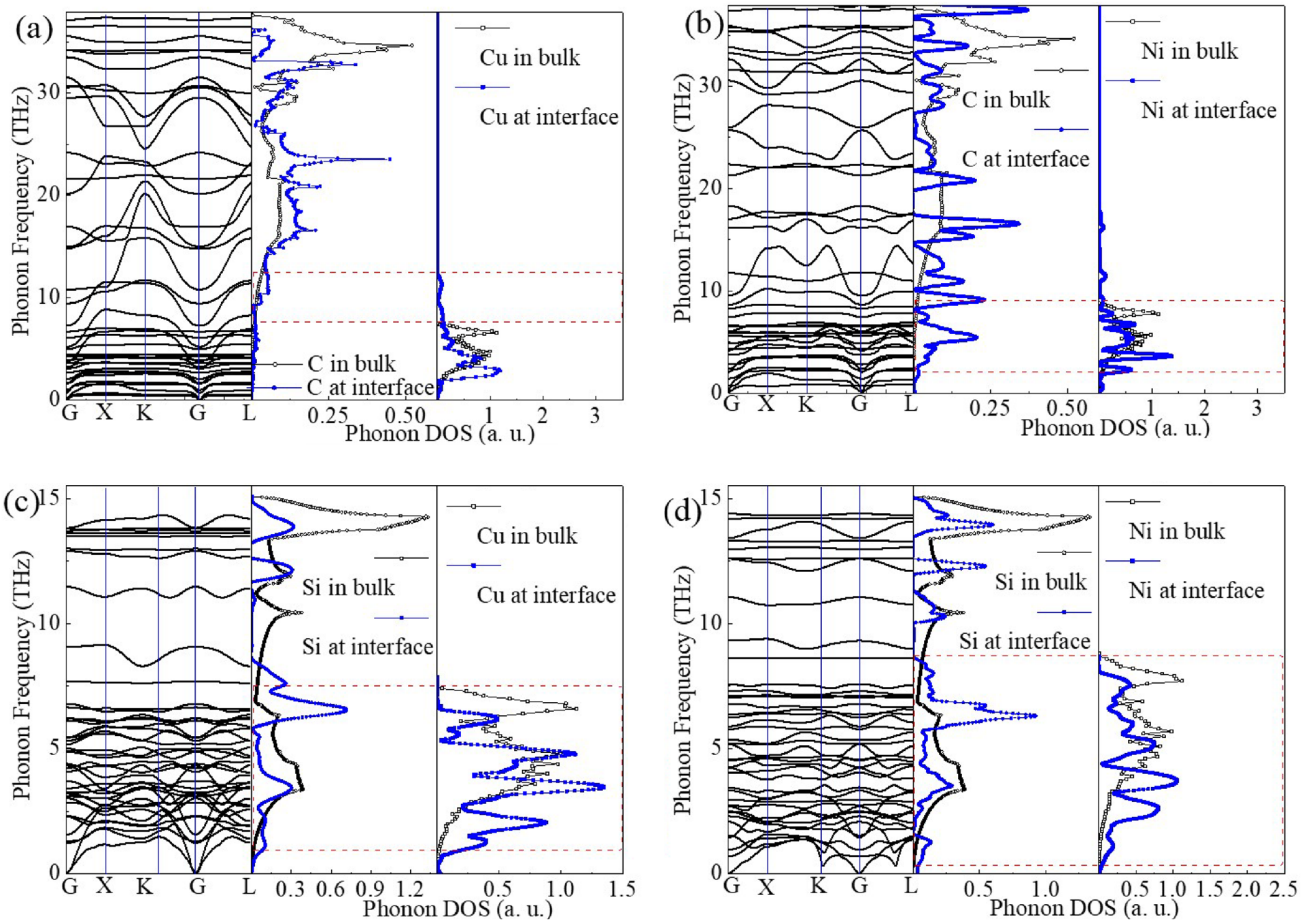
(**a**) Phonon dispersion curves and phonon DOS of the interfacial atoms for (**a**) the Cu–C, (**b**) the Ni–C, (**c**) the Cu–Si, (**d**) the Ni–Si interfaces. The phonon DOS of the atoms in bulk are also shown.

In order to further study the phonon coupling at these interfaces in detail, the phonon dispersion of these interfaces were calculated and displayed in Fig. 4a–d. In addition, the projected phonon DOSs for each Cu, Ni, C and Si atoms at these interfaces were also calculated to clarify the contribution of the interfacial atoms to the total phonon DOS as shown in Fig. 4.

For Cu–C interface in Fig. 4a, compared to the C–C covalent bond in bulk diamond and the Cu–Cu metal bond in bulk Cu, the interaction between C and Cu near the copper/diamond interface is obviously different. Moreover, the dimerization reconstruction of surface atoms could inevitably occur on the surface of semiconductors due to the very weak interaction between metals and semiconductors. The redistribution of the phonon for C in the side of diamond has occurred near the interface, and the phonon peaks have moved towards the position of lower frequencies. And in the side of Cu, the phonon DOSs of Cu in the region of interface has also been reconstructed to a certain extent, and a certain amount of new vibration modes are produced in the higher frequency.

Similar phonon reconstitution of the interfacial atoms also occurs at the other interfaces as shown in Fig. 4b–d. The overlap of the phonon DOS for these interfacial atoms has been shown by a red dashed box in Fig. 4a–d. It can be seen from the overlap of the phonon DOSs that the coupling strength of the phonon is relatively lower at the Cu–C interface as shown in Fig. 4a. Besides, because the strain is imposed on the metal, the phonon spectral will shift toward the higher frequencies to a certain extent as shown in Fig. S2 in Supplementary Material. And the phonons in the metals are blue-shifted to higher frequencies, which will increase the overlap of the density of phonon states between metal and semiconductor. Usually, the larger overlapping DOS between the metals and semiconductors could be considered to provide more for phonons scattering channels to transport across the interface.

**Figure 5:**
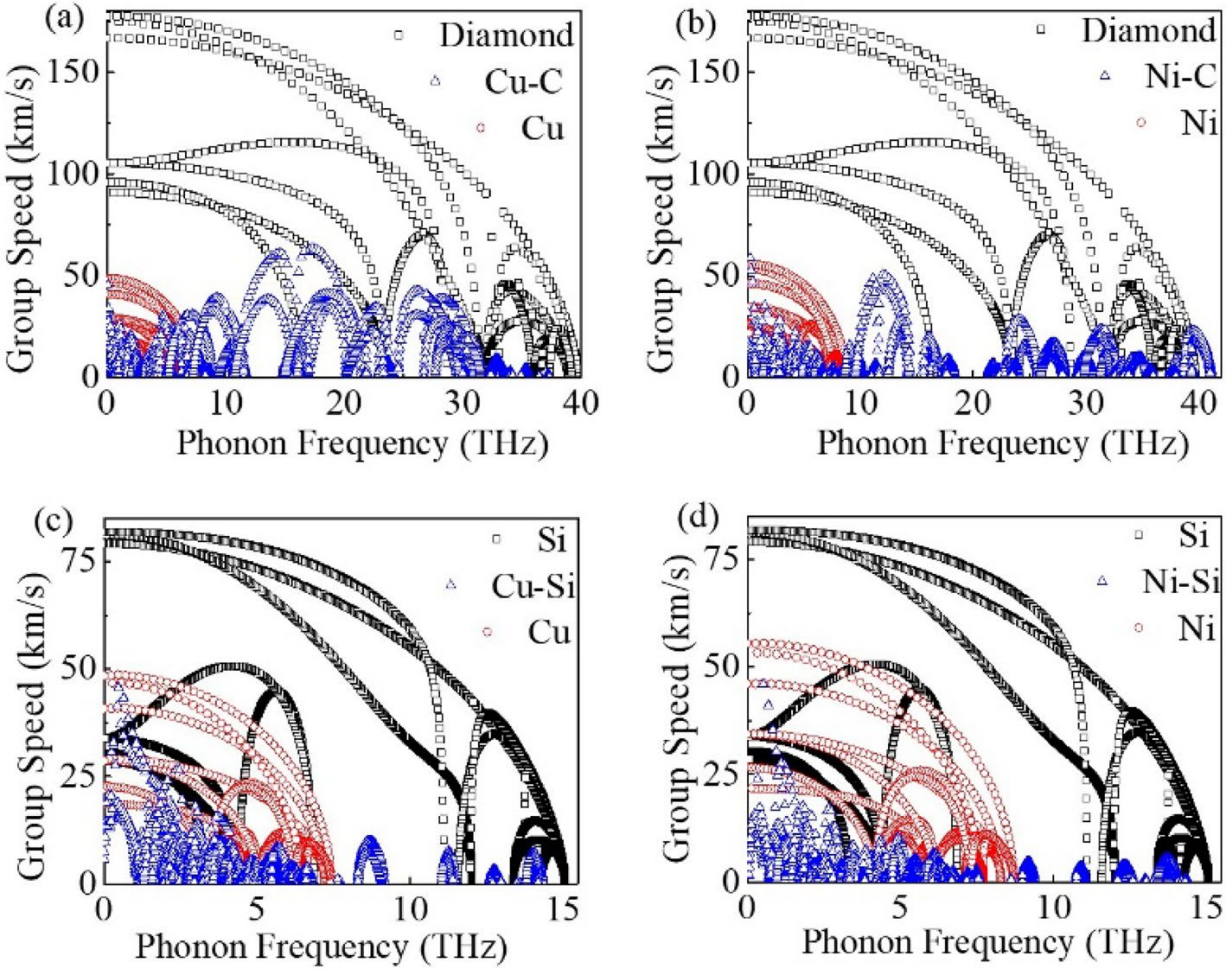
Phonon group velocities with respect to phonon frequency for (**a**) the Cu–C, (**b**) the Ni–C, (**c**) the Cu–Si, (**d**) the Ni–Si interfaces. Phonon group velocities for the bulk Cu, the bulk Ni, the bulk diamond and the bulk Si are also shown.

According to Eqs. (3) and (5) for the transmission of phonons $\alpha_{1 \to 2}$ and G, the phonon group velocities is the other critical parameter. Accurate calculation of G is a complex subject with server mechanisms involved, such as coupling between phonons, electrons in metals and phonons in semiconductors, phonons interacted by electrons in metals and phonons in semiconductors. In this work, phonon is only considered to be the hot carriers, and the coupling between phonons is only involved. Then, the frequency-dependent phonon group velocities of the metals, semiconductors and various interfaces were calculated and shown in Fig. 5a–d.

The results show that the group velocities of the interfaces based on Si are significantly lower than those of the interfaces based on diamond, and the group velocities of the interfaces based on Si are mainly distributed in the relatively low frequency (below 10 THz) as shown in Fig. 5c,d, and the group velocities of the interfaces based on diamond are scattered below 40 THz and disappears in the relatively high frequency above 40 THz.

**Figure 6:**
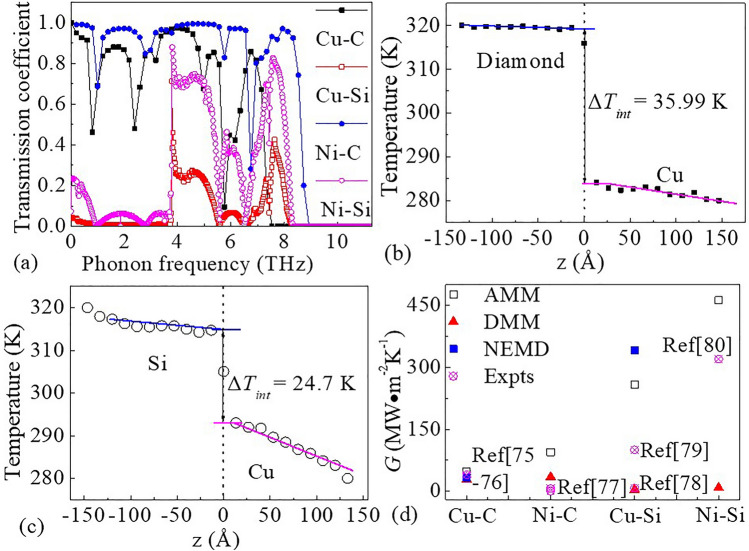
(**a**) Phonon transmission coefficients for the Cu–C, Ni–C, Cu–Si and Ni–Si interfaces. Temperature profile for the (**b**) Cu–C and (**c**) Cu–Si along the direction of z at room temperature. (**d**) Interface thermal conductance (*G*) for the Cu–C, Ni–C, Cu–Si and Ni–Si interfaces. The experimental data from Refs.^75–80^ are also shown for comparison.

For the convenience of comparisons of simulation results, the phonon transmission from the DMM model and the temperature jump (ΔT) from the NEMD were shown in the Fig. 6a–c, respectively. And the calculated interfacial thermal conductance and their experimental value were also shown in Fig. 6d. According to the phonon dispersions and phonon DOSs calculated for Cu, Ni, diamond and Si, and Eq. (3) for the phonon transmission, the relationship between phonon transmission and phonon frequency can be obtained and shown in Fig. 6a. At the lower frequency (less than 10 THz), phonon transmission coefficients are showing an oscillating characteristic with the increasing frequency, which can be mostly attributed to periodic mass distribution^24^. Besides, it can also be seen from the Fig. 6a that the phonon transmission is quite high at the lower frequency (less than 7.4 THz for Cu–C and 8.9 THz for Ni–C). It is due to the fact that phonons with small frequencies have larger wavelengths and can pass through interfaces based on the diamond more easily^74^. While for interfaces based on the Si, the phonon transmission is quite high in the frequency range of 3.8–8.2 THz. According to Eq. (5), a higher phonon transmission at interface would lead to an increase of the *G*. According to the Eq. (5) for computing the *G*, the values of *G* for the Cu–C, Ni–C, Cu–Si and Ni–Si interfaces can be calculated to be 27.87, 34.13, 3.64 and 8.41 MW m^−2^ K^−1^ at room temperature, respectively.

In addition, for AMM the *G* of the interface can be calculated using a simple Debye model given as^20^:

6 $$ G = C_{1} \cdot \rho_{1} \cdot {\text{V}}_{{1}}^{{3}} \cdot \left( {\rho_{1} {\text{V}}_{{1}} \rho_{2} {\text{V}}_{{2}} } \right)/\left( {{\text{ 2V}}_{{2}}^{{2}} \cdot \left( {\rho_{1} {\text{V}}_{{{1} + }} \rho_{2} {\text{V}}_{{2}} } \right)^{{2}} } \right), $$

where *C*_1_ is the specific heat of metal, *ρ*_1_, *ρ*_2_, V_1_ and V_2_ are the density and the sound velocity of metals and semiconductors, respectively. And sound velocity is given by 3/V^2^ = 1/V_L_^2^_+_2/V_T_^2^, where longitudinal and transverse sound velocities denoted by V_L_ and V_T_, respectively. These important characteristic parameters^20^ of materials adopted were listed in Table 3. According to the Eq. (6), the values of *G* for the Cu–C, Ni–C, Cu–Si and Ni–Si interfaces can be calculated to be 47.6, 94.1, 258.33 and 462.32 MW m^−2^ K^−1^ at room temperature, respectively. The obtained values of *G* using the DMM and AMM are shown in Fig. 6d.

**Table 3:** Relevant characteristic parameters of various materials used to calculate interfacial thermal conductivity.

Materials	Density (kg m^−3^)	V_L_ (km s^−1^)	V_T_ (km s^−1^)	V (km s^−1^)	Specific heat (J K^−1^ kg^−1^)	Thermal conductivity (W m^−1^ K^−1^)
Ni	8880	5.630	2.960	3.398	444	88
Cu	8900	4.910	2.500	2.8808	385	397
Si	2330	8.970	5.332	6.0202	703	126
Diamond	3520	17.500	12.800	13.925	508	1800

Longitudinal sound velocity, Transverse sound velocity and sound velocity are abbreviated as V_L_, V_T_ and V, respectively. The experimental data are from Ref.^20^.

For NEMD, the *G* of every interface was calculated according to the equation^81^

7 $$ G = J/ \, ({\text{A}} \cdot \Delta {\text{T}}), $$

where* J* represents the average heat flow along the temperature gradient (dQ/dt), A is the cross-sectional area perpendicular to the direction of heat flow, and ΔT is the temperature jump.

After a steady state reached, the typical temperature profiles for Cu–C and Cu–Si heterostructure were shown in Fig. 6b,c, respectively. According to the temperature profiles, the values of ΔT were obtained to be 35.99 K and 24.7 K at Cu–C and Cu–Si interfaces by extrapolating the linear fit of the temperature, respectively. Besides, the heat flux *J* was recorded during the MD simulation through the energy change rate in the local heat treatment, and finally the *G* can be computed according to Eq. (7). And the values of *G* were obtained to be 32.55 MW m^−2^ K^−1^ and 341.87 MW m^−2^ K^−1^ at Cu–C and Cu–Si interfaces, respectively. As a comparison, the values were also shown in the Fig. 6d.

From Fig. 6d, the data points are significantly distant, indicating that the values of *G* have a noticeable difference through the DMM and AMM. From a numerical point of view, in comparison with the values of *G* calculated by AMM, the values of *G* calculated through DMM are much smaller. Then compared with the calculation results from NEMD method, the values of 32.55 MW m^−2^ K^−1^ for Cu–C interface is closer to the result (27.87 MW m^−2^ K^−1^) from DMM while the values of 341.87 MW m^−2^ K^−1^ for Cu–Si interface is closer to the result (258.33 MW m^−2^ K^−1^) from AMM. These results of theoretical simulation calculations are basically consistent with the current experimental data, which indicates that phonon–phonon interaction play a more important role than electron–phonon interaction during heat transport. Another important reason is mainly because the experimental data is also relatively scattered as shown in Fig. 6d, for example, 29.7–40 MW m^−2^ K^−1^ for the Cu–C^75,76^, 6.5–6.7 MW m^−2^ K^−1^ for Ni–C^77^, 12.5–100 MW m^−2^ K^−1^ for the Cu–Si^78,79^ and 345 MW m^−2^ K^−1^ for the Ni–Si interfaces^80^. However, some experimental values are not in good agreement with the predicted values from the theoretical simulation calculations, which may be due to its strong dependence on the preparation conditions^78^.

According to the calculation results using DMM, we found that the *G* of the interface based on diamond is obviously larger, while the results from AMM show that the *G* of the interface based on silicon is significantly larger. This is mainly due to the very important role the theoretical basis of the DMM and AMM is playing in the calculation for the *G* of the interfaces. In the AMM^20,23^, phonons transport across the interface by reflection and refraction at the interface, so they are more suitable for long-wave phonons. In the DMM^82^, phonons transport across the interface entirely in diffuse scattering at the interface, where phonon transmission is connected with the mismatch of the phonon DOS of the metal and semiconductor. Therefore, the DMM can better describe the phonon transport at the interface at room temperature^83^. Besides, according to the calculation of *G* by Keblinski^84^, it is shown that the value of *G* is also very sensitive to the roughness and disorder of the interface. Considering the higher interface coupling strength, the calculation results of the *G* using DMM also show that the *G* of the interface based on diamond is significantly larger. Although the thermal conductance of materials or interfaces is easily affected by many factors^85^, it may be effective way to improve the interfacial thermal conductance through enhancing the interface coupling strength at the metal–semiconductor interface. One of the reasons for this is that strong interfacial scattering plays a role in suppressing most of phonon modes in the weaker interface coupling heterostructure, leading to the lower *G*^86,87^.

## Conclusion

The formation energy and electronic properties of the interfaces Cu–C, Cu–Si, Ni–C and Ni–Si were calculated by the first principle method. Then the *G* for these interfaces were performed by DMM and AMM. Especially, the *G* for Cu–C and Cu–Si interfaces was further validated by NEMD method. The results show that the absolute value of interface formation energy increases when C is changed to Si, from − 1.88 eV for Cu-C to − 0.23 eV for Cu–Si interface, and decreases when C is changed to Si, from − 0.45 for Ni–C to − 0.17 eV for Ni–Si interface. In addition, the values of Schottky barrier are 1.57, 1.45, 0.66 and 0.61 eV for the Cu–C (p type), Ni–C (p type), Cu–Si (n type) and Ni–Si (n type) interfaces, respectively. For thermal conductance of interface at room temperature, according to NEMD method, the values of *G* were obtained to be 32.55 MW m^−2^ K^−1^ and 341.87 MW m^−2^ K^−1^ at Cu-C and Cu-Si interfaces, respectively. The values of the *G* are 27.87, 34.13, 3.64 and 8.41 MW m^−2^ K^−1^ for the Cu–C, Ni–C, Cu–Si and Ni–Si interfaces by DMM, respectively. For AMM, the values of *G* are 47.6, 94.1, 258.33 and 462.32 MW m^−2^ K^−1^for the Cu–C, Ni–C, Cu–Si and Ni–Si interfaces, respectively. These results of theoretical simulation calculations are basically consistent with the current experimental data, which indicates that phonon–phonon interaction play a more important role than electron–phonon interaction during heat transport. It could be an effective way to increase the phonon transmission through increasing the interface coupling strength, because strong interfacial scattering plays a role in suppressing most of phonon modes in the weaker interface coupling heterostructure. The findings in the work will be conducive to understand the existing of the other channels in the process of thermal transport apart from phonon.

## Supplementary Information

Supplementary Figures.

## Data Availability

THE authors declare that all relevant data supporting the findings of this study are available from the corresponding authors on request.
