# Supplementary material for: A comparative study of interfacial thermal conductance between metal and semiconductor
Source: Sci Rep. 2022 Nov 19;12:19907. doi: 10.1038/s41598-022-24379-z (PMC9675788; doi:10.1038/s41598-022-24379-z)
Supplement: Supplementary file 1 — Supplementary Figures. [file 41598_2022_24379_MOESM1_ESM.docx]

**A comparative study of interfacial thermal conductance between metal and semiconductor**

Kongping Wu^[[1]](#footnote-1),†^, Leng Zhang^1^, Danbei Wang^1^, Fangzhen Li^1^, Pengzhan Zhang^1^, Liwen Sang^2^, Meiyong Liao^[[2]](#footnote-2),‡^, Kun Tang^3^, Jiandong Ye^3^, Shulin Gu^3^

^1^School of Electronics and Information Engineering, Jinling Institute of Technology, Nanjing Jiangsu, 211169, China

^2^Research Center for Functional Materials, National Institute for Materials Science (NIMS), Tsukuba Ibaraki, 305-0044, Japan

^3^School of Electronic Science and Engineering, Nanjing University, Nanjing Jiangsu, 210093, China

Fig. S1 The total energy of Cu, Ni, Si and diamond as a function of lattice constants.


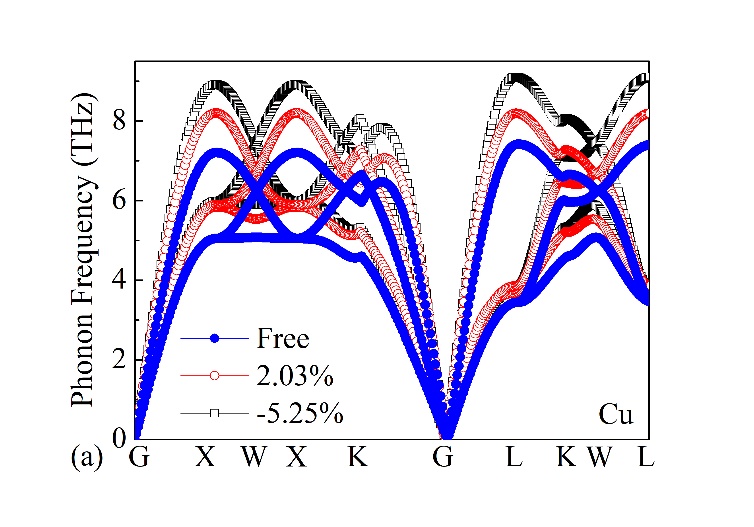

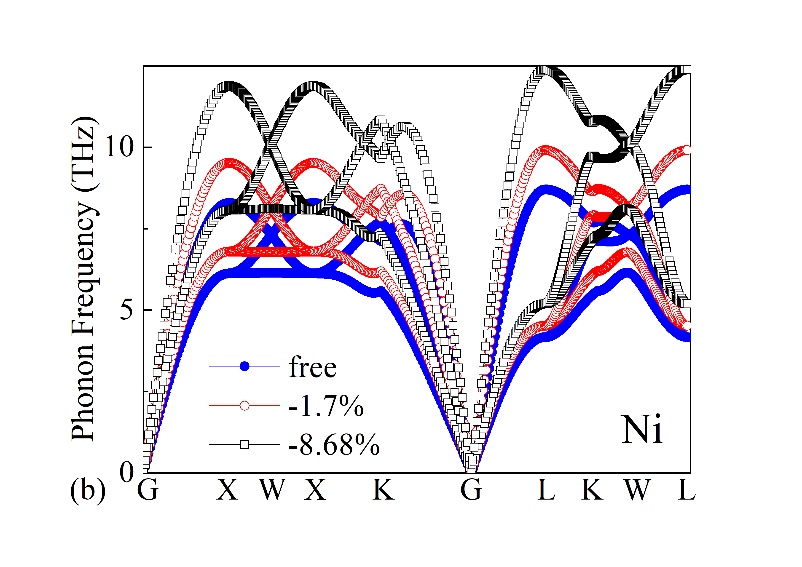


Fig. S2 The effect of strain on the phonon spectral of the metal Cu (a) and Ni (b).

1. ^†^ Corresponding author. Tel: +86(025) 8618-8572. E-mail address: [kpwu@jit.edu.cn](mailto:kpwu@jit.edu.cn) (K. P. Wu). [↑](#footnote-ref-1)
2. ^‡^ Corresponding author. Tel: +81(29) 860-4508. E-mail: [Meiyong.Liao@nims.go.jp](mailto:Meiyong.Liao@nims.go.jp) (M. Y. Liao). [↑](#footnote-ref-2)
